# The clinical burden of human cystic echinococcosis in Palestine, 2010-2015

**DOI:** 10.1371/journal.pntd.0005717

**Published:** 2017-07-03

**Authors:** Amer Al-Jawabreh, Suheir Ereqat, Kamal Dumaidi, Abdelmajeed Nasereddin, Hanan Al-Jawabreh, Kifaya Azmi, Nahed Al-Laham, Moath Nairat, Adriano Casulli, Husni Maqboul, Ziad Abdeen

**Affiliations:** 1 Department of Medical laboratory Sciences, Faculty of Allied Health Sciences, Arab American University, Jenin, Palestine; 2 Al-Quds Public Health Society, Jerusalem, Palestine; 3 Biochemistry and Molecular Biology Department, Faculty of Medicine, Al-Quds University, Abu Deis, East Jerusalem, Palestine; 4 Al-Quds Nutrition and Health Research Institute, Al-Quds University, East Jerusalem, Palestine; 5 Department of Laboratory Medicine, Faculty of Applied Medical Sciences, AlAzhar University–Gaza, Gaza Strip, Palestine; 6 Palestine Medical Complex, Ministry of Health, Ramallah, Palestine; 7 World Health Organization Collaborating Centre for the Epidemiology, Detection and Control of Cystic and Alveolar Echinococcosis (in humans and animals), Istituto Superiore di Sanità, Rome, Italy; 8 European Union Reference Laboratory for the Parasites, Istituto Superiore di Sanità, Rome, Italy; 9 Faculty of Medicine and Health Sciences, An-Najah National University, Nablus, Palestine; St. George's University, GRENADA

## Abstract

**Background:**

Cystic echinococcosis (CE) is classified by the WHO as a neglected disease inflicting economic losses on the health systems of many countries worldwide. The aim of this case-series study was to investigate the burden of human CE in Palestine during the period between 2010 and 2015.

**Methodology/Principal findings:**

Records of surgically confirmed CE patients from 13 public and private hospitals in the West Bank and Gaza Strip were reviewed. Patients’ cysts were collected from surgical wards and formalin-fixed paraffin-embedded (FFPE) blocks were collected from histopathology departments. Molecular identification of CE species /genotypes was conducted by targeting a repeat DNA sequence (EgG1 Hae III) within *Echinococcus* nuclear genome and a fragment within the mitochondrial cytochrome c oxidase subunit 1, (CO1). Confirmation of CE species/genotypes was carried out using sequencing followed by BLAST analysis and the construction of maximum likelihood consensus dendrogram. CE cases were map-spotted and statistically significant foci identified by spatial analysis. A total of 353 CE patients were identified in 108 localities from the West Bank and Gaza Strip. The average surgical incidence in the West Bank was 2.1 per 100,000. Spot-mapping and purely spatial analysis showed 13 out of 16 Palestinian districts had cases of CE, of which 9 were in the West Bank and 4 in Gaza Strip. Al-Khalil and Bethlehem were statistically significant foci of CE in Palestine with a six-year average incidence of 4.2 and 3.7 per 100,000, respectively.

**Conclusions/Significance:**

To the best of our knowledge, this is the first confirmation of human CE causative agent in Palestine. This study revealed that *E*. *granulosus* sensu stricto (s.s.) was the predominating species responsible for CE in humans with 11 samples identified as G1 genotype and 2 as G3 genotype. This study emphasizes the need for a stringent surveillance system and risk assessment studies in the rural areas of high incidence as a prerequisite for control measures.

## Author summary

Cystic echinococcosis (CE) is a neglected disease caused by a parasite called *Echinococcus granulosus*. The dog as a definitive host plays a major role in transmitting the infection to human. The study aimed to investigate the clinical burden of human CE in Palestine during the period between 2010 and 2015. Thirteen hospitals in the West Bank and Gaza Strip were targeted. CE patients’ records were reviewed. Patients’ samples were collected including cysts following surgery and formalin-fixed paraffin-embedded (FFPE) blocks from histopathology departments. Molecular identification of CE species /genotypes was conducted by targeting nuclear and mitochondrial DNA and confirmed by identifying the DNA sequence and comparing it with those in the Genebank. CE cases were spot-mapped and statistically significant foci identified. A total of 353 CE patients were identified in 108 localities in Palestine from the West Bank and Gaza Strip. The average surgical incidence in the West Bank was 2.1 per 100,000 with Al-Khalil district reporting the highest incidence in Palestine. This study revealed that *E*. *granulosus* sensu stricto (s.s.), G1 genotype was the main species responsible for CE in humans. There is need for a surveillance system and risk assessment studies in the rural areas as a prerequisite for control measures.

## Introduction

Cystic Echinococcosis (CE) is a zoonotic parasitic infection caused by the metacestode larval stage of species within the genus *Echinococcus*. CE as the most frequently encountered disease is caused by *Echinococcus granulosus* sensu lato (s.l.) with the dog as the main definitive host and ungulates, mainly sheep as the intermediate host. Humans are accidental hosts infected following the ingestion of eggs shed in dog faeces. Although the classification and taxonomy of the genus *Echinococcus* is still controversial, however recent classification recognizes nine species, five of which belong to *E*. *granulosus* sensu lato (s.l.) namely *E*. *granulosus* sensu stricto (s.s.) (genotypes G1-G3), *E*. *felidis* (lion strain), *E*. *equinus* (genotype G4), *E*. *ortleppi* (genotype G5), and *E*. *canadensis* (genotypes G6/7-G8 and G10) [1, 2]. CE is considered by WHO as an important food-borne parasitic disease with estimated 1–3 million Disability adjusted life years (DALYs) for cystic echinococcosis (accounting for underreporting), [3]. CE is common worldwide with hyperendemic areas exceeding 50 cases per 100,000 in some countries like Argentina, Peru, East Africa, Central Asia, and China and causing annual loss of approximately $3 billion, yet still listed as one of the 18 neglected diseases in the world [4–6]. In the Mediterranean region where CE is endemic, Tunisia and Morocco reported the highest incidence of 12.5 and 5.1 surgical cases per 100,000, respectively [7–12]. In Turkey, an ultrasonography-based survey among children revealed a prevalence of 0.2% with *E*. *granulsus* sensu stricto (s.s.) (G1/G3) as the main cause [13, 14]. Historical records in Palestine put the disease incidence at 1 and 5/100,000 during 1922–1935 and 1959, respectively [15, 16]. More recently, the incidence of human CE in 2015 was officially reported to be 1.6 per 100,000 [17]. This rate depends exclusively on surgical incidence reported by government hospitals following surgery. Pilot studies showed that the incidence rate in dogs as definitive hosts using copro-PCR was 18% [18]. The most common genotype in the definitive (dog) and intermediate hosts (sheep) from Palestine is *E*. *granulosus* G1 genotype [18, 19]. In adjacent areas like the city of Rahat and Bir-Al Saba’, CE was predominant among Bedouin community compared to Jewish residents with an incidence of 2.7 and 0.4 per 100,000, respectively [20].

In this study we aimed to investigate the clinical burden of human cystic echinococcosis in Palestine (West Bank and Gaza Strip) using retrospective hospital records during the period between 2010 and 2015 supported by molecular methods through amplification of DNA from human cysts and formalin-fixed paraffin embedded (FFPE) blocks. In this study surgical incidence was defined as the frequency of operated CE cases per 100,000 inhabitants per year.

## Materials and methods

### Ethics statement

This study was ethically approved by the Ministry of Health (MoH) in Palestine (162/2044/2015). All patients’ data were securely archived and anonymized.

### Study design, medical records and sample collection

A case series observational study on surgically-confirmed human CE cases in Palestine (The West Bank and Gaza Strip) was carried out during a six year period between 2010 and 2015.

CE patients’ records were retrieved from public (government) and private hospitals in the West Bank and Gaza Strip. All reviewed CE cases had been diagnosed using ultrasonography or computed tomography (CT) and histopathologically confirmed following surgery. Thirteen hospitals were included in the study namely Jenin Government Hospital, Al-Amal Hospital (Jenin), Zakat Hospital (Jenin), Rafidia Government Hospital in (Nablus), Al-Khalil Government Hospital, Al-Ahli Hospital in Al-Khalil, Al-Mezan Hospital in Al-Khalil, Beit-Jala Government Hospital in Bethlehem, Jericho (Ariha) Government Hospital, Ramallah Government Hospital, Al-Makassed Hospital in East Jerusalem (Al-Quds), Al-Shifa Government hospital in Gaza, and Gaza European Hospital in Rafah. This included all government hospitals and major private hospitals that serve a population of ca. 4.6 million Palestinians in the West Bank and Gaza Strip. Retrieved data included patients’ demography such as names, age, address, date of birth, sex, site of infection, and diagnosis based on histopathology reports. Missing demographic and clinical data were obtained by conducting phone interviews with patients facilitated by the Ministry of Interior in Palestine. Human CE material removed from surgically-confirmed patients was collected from the histopathology departments in the form of FFPE pathology blocks. In addition, CE cysts surgically-removed during the duration of this study were provided by the relevant wards and stored at -20°C.

### Molecular assays

#### Genomic DNA extraction from frozen cyst tissue

Frozen CE tissue samples were thawed at room temperature and approximately 0.03g of tissue was cut with a sterile blade into pieces which were then placed into a 1.5 ml Eppendorf tube. Five hundred μl of lysis buffer (10 mM Tris-HCl (pH 7.4), 10 mM EDTA, 50 mM NaCl, 0.5% sodium dodecyl sulfate, and 20 mM dithiothreitol or 0.2% (v/v) 2-mercaptoethanol) were then added and the samples were incubated at 95°C for 10 min on a thermal block (Eppendorf^®^ Thermomixer^®^) with continuous shaking at 500 rpm. This was followed by the addition of 0.5 mm glass beads (Cell disruption media, Scientific Industries, Inc-SI.BG05) to each tube with constant shaking for 5 minutes at 2,850 rpm using disruptor Genie (Scientific industries, Inc) until the tissue was completely lysed. Proteinase K (500μg/ml) was added to the lysed samples which were then vortexed vigorously for 5 seconds and incubated overnight at 56°C under constant agitation at 450 rpm on the thermal block. Proteinase K was inactivated at 98°C for 8 min. DNA was extracted using phenol-chloroform, followed by the addition of 2% CTAB extraction buffer for 10 min (2% cetyl trimethylammonium bromide, 3% polyvinyl pyrrolidine (PVP), 0.2% 2-mercaptoethanol, 100mM Tris-HCl, 1.4 M NaCl, 20 mM EDTA) and followed with a phenol-chloroform step. Total genomic DNA was ethanol-precipitated and the pellet was re-suspended in 900 μl of Guanidine Thiocyanate (GuSCN) lysis buffer (60% w/v GuSCN, 50 mM Tris-HCl, pH 6.4, 22 mM EDTA, and 1.2% v/v Triton X-100) and thoroughly mixed. Forty microliters of diatomaceous earth suspension (20% w/v in 1% HCl) were then added and incubated for 10 min. Two washes using GuSCN buffer (60% w/v GuSCN, 50 mM Tris-HCl) and a further two washes with 70% ethanol were performed, followed by centrifugation at max speed for 5 min. After drying the ethanol, DNA was eluted in 50 μl TE buffer (10 mM Tris and 1 mM EDTA pH 7.5) and incubated at 56°C for 10 min [21–23]. DNA concentration and quality, (A_260_/A_280_) ratio were measured using a spectrophotometer (Nanodrop 2000C, Thermo Fisher Scientific-USA) and by visualization on an agarose gel, respectively.

#### Genomic DNA extraction from FFPE samples

Around 7–10 ribbon sections for each CE sample were cut using a rotary microtome at a thickness of 10μm and placed in 1.5 ml microfuge tubes. The blade was cleaned with ethanol in between each sample to prevent carry over contamination. Deparaffinization was carried out by adding 1 ml of xylene, and incubating the sample for 10 minutes at 37°C on a thermal heat block (Eppendorf^®^ Thermomixer^®^) with continuous shaking at 400 rpm. The sample was vigorously vortexed for 10 seconds, and centrifuged at 13,000 rpm for 5 minutes. The supernatant was aspirated without disturbing the pellet and the procedure was repeated one more time. Xylene was removed using a series of gradient ethanol washes, 96%, 70%, and 50% and centrifuged for 5 min at 13,000 rpm. DNA extraction was processed as described above.

#### Amplification of DNA and Genotyping of CE

The molecular confirmation of retrospective CE infection using DNA extracted from both FFPE blocks and CE cysts was accomplished through the amplification of a tandem repeat unit (269bp) within the nuclear genome of *Echinococcus* species [17]. The identification of *Echinococcus* species and/or genotypes was carried out through the amplification of a fragment (440bp) within the mitochondrial cytochrome *c* oxidase subunit 1 [16] using DNA extracted from cyst samples only. Amplified products were sequenced from both directions. Generated sequence data was visualized using MEGA 7 and compared with that present on GenBank database through the use of BLAST algorithm (http://www.ncbi.nlm.nih.gov/BLAST/).

### Statistical analysis

Kulldorff’s SaTScan programme v9.4.3 was used to assign spatial and space-time distribution of cases in Palestine based on number of cases per locality, year of diagnosis, population size of locality at time of diagnosis, and the exact latitude-longitude coordinates of each location. Data were analyzed based on a discrete Poisson model with the level of statistical significance considered at P-value ≤ 0.05 [24]. Spot mapping of cases and frequencies were analyzed using Epi Info statistical package (CDC free-software). The level of statistical significance was considered at P-value ≤ 0.05. Evolutionary analysis, genetic relationship, and multiple alignments were conducted in MEGA 7 [25].

## Results

### Demographic distribution

**Fig 1:**
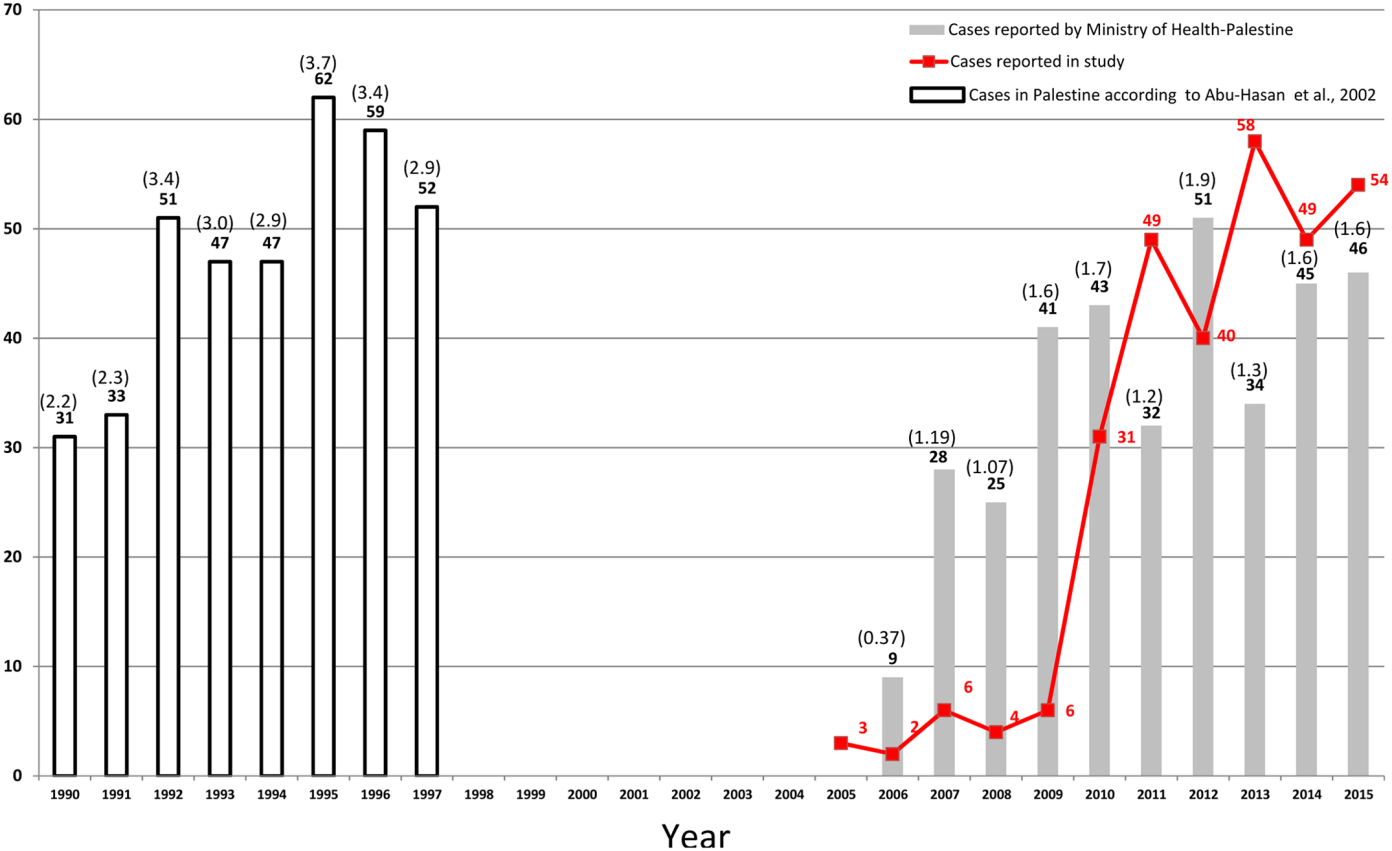
Cases of human cystic echinococcosis (CE) recorded between 1990 and 2015 in Palestine. The bar graph in grey represents the official figures for CE cases registered by the Palestinian Ministry of Health (MoH). The blank bar graphs are after Abu-Hasan and colleagues [26]. The numbers in brackets are the incidence rates per 100,000 in the West Bank and the numbers below them represent the total number of cases reported per annum. The red line graph represents the numbers of CE cases collected in the study and known for their year of surgery.

**Fig 2:**
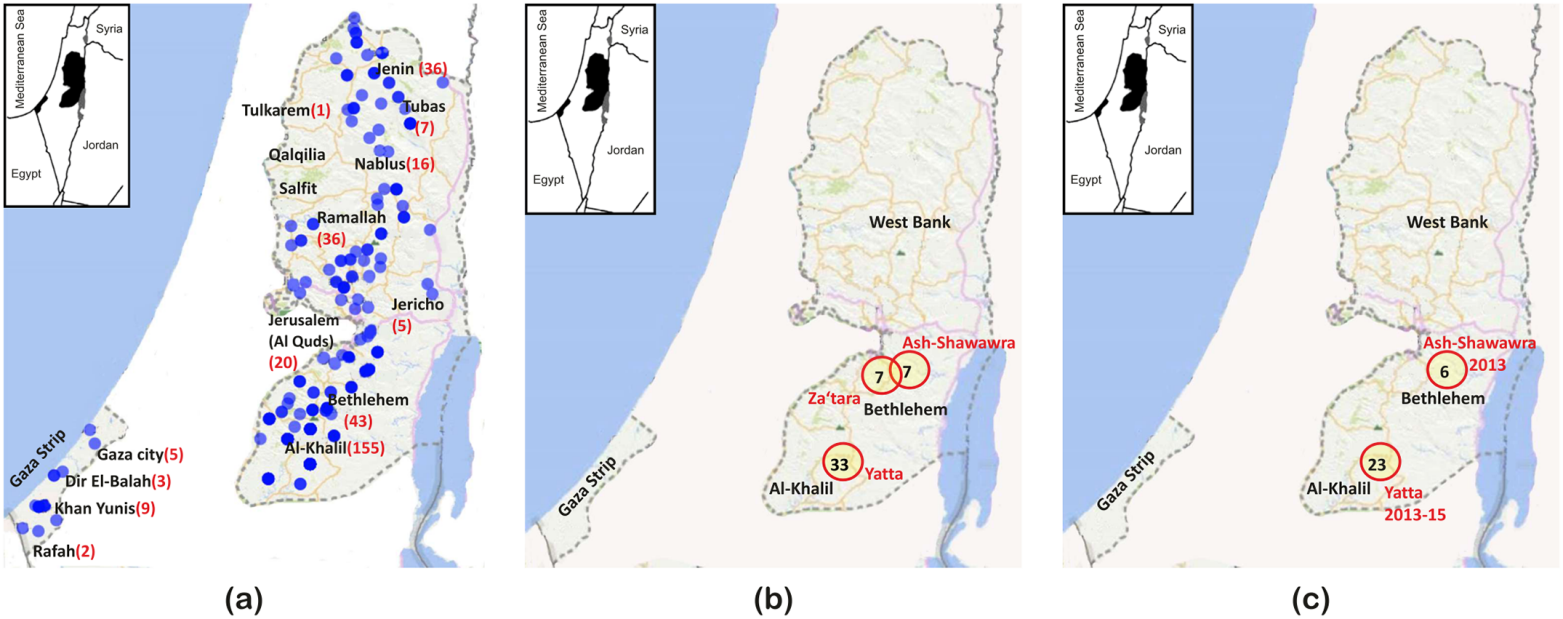
Distribution of human cases of cystic echinococcosis in Palestine. (a) Spot mapping of 338 cases (blue circles) based on actual lat-long coordinates with light blue circles representing one case while dark blue circles representing more than one case in some areas. Major Palestinian cities are shown (b) Purely-spatial distribution of 268 cases based on SaTScan software showing three major statistically-significant (P<0.05) foci, each represented by number of cases within a circle. (c) Space-time distribution of 268 cases showing two statistically-significant foci, each represented by number of cases within a circle.

During 2010–2015 a total of 353 CE patients were identified in 13 hospitals in the West Bank and Gaza Strip, the vast majority of whom (n = 282) were diagnosed during this study period (Fig 1). Cystic echinococcosis was reported in 108 localities in the West Bank (94%, 319/338) and Gaza Strip (6%, 19/338) (Fig 2a). The average surgical incidence for the disease in the West Bank, Gaza Strip, and Palestine as a whole was approximately 2.1, 0.13, and 1.1 per 100,000, respectively. Demographic habitats of CE cases were shown to include villagers (78%), city-dwellers (20%), refugee camp residents (2%) and Bedouins living in encampments (0.3%). The female-to-male ratio was 1.13 (187:166) which was not statistically significant (Chi square = 1.2, P = 0.26). The age of CE patients ranged from 2 to 86 years old, with the two age groups 10–19 and 20–29 having significantly high number of reported hydatid cyst cases than expected (Chi square = 145.5, P = 0.0001). After this, CE cases decreased gradually until reaching 4 in the age group 80–89 years (Table 1).

**Table 1:** The distribution of CE cases by age group and sex.

Age group	Total	Male	Female
0–9	44	24	20
10–19	68	37	31
20–29	73	35	38
30–39	56	23	33
40–49	44	17	27
50–59	23	9	13
60–69	11	7	4
70–79	8	4	5
80–89	4	3	1
	**331**	**159**	**172**

### Distribution of cases, time and space

Human cases of CE started to appear systematically on the Palestinian Ministry of Health (MoH) annual report from 2006 with a peak in 2012. The MoH annual report also reported Gaza Strip as CE-free area except for 1 case in 2011, while this study revealed 19 cases in the same period. Of the 353 CE cases, the residential premises of 338 were known and were therefore used for spot-mapping of CE cases. CE was found to be present in thirteen out of 16 Palestinian districts, 9 regions of which were in the West Bank and 4 in the Gaza Strip (Fig 2a). District-wise, Al-Khalil and Bethlehem were the main foci of CE in Palestine with a six-year average incidence of 4.2 and 3.7 per 100,000, respectively. Purely spatial analysis identified villages of Yatta in Al-Khalil District and Z’atara, and Ash-shawawra in Bethlehem District to be statistically significant foci for the disease and the most prevalent urban areas in Palestine with an annual CE incidence of 9.6, 13.9, and 23.3 per 100,000, respectively (Fig 2b). On the other hand, in the space-time analysis two foci were spotted in certain years over the study period, one in Al-Khalil and another in Bethlehem (Fig 2c).

### Clinical manifestation

Of the 261 CE patients, information regarding localization of CE infection was known for 271 cysts. Liver cysts were exclusively found in 158 (58%) of CE cases whereas pulmonary infection was recorded in 27% (74/271) of cases. Approximately 3.4% (9/261) of CE cases had multiple site infection with two or more organs being involved (Table 2). Of the 299 cases for which symptoms were known, the most frequent symptoms were abdominal pain (42%) and dyspnea (13%) (Table 3).

**Table 2:** Distribution of site of infection of CE cases in Palestine, 2010–2015.

Organ(s)	No. CE case	No. CE cyst (%)
Liver	158	158 (58)
Lung	74	74 (27)
Abdomen	5	5(1.8)
Liver and lung	4	8 (3)
Liver and spleen	2	4 (1.5)
Spleen	3	3 (1)
Kidney	2	2 (0.7)
Gallbladder	1	1 (0.4)
Ovary	1	1 (0.4)
Lung and brain	1	2 (0.7)
Muscles of the back	1	1 (0.4)
Axillary region	1	1 (0.4)
Leg	1	1 (0.4)
Testis	1	1 (0.4)
Liver and brain	1	2 (0.4)
Liver, Pelvis, and Omentum	1	3 (1)
Sacroiliac joint	1	1 (0.4)
Brain	1	1 (0.4)
Bone	1	1 (0.4)
Intestines	1	1 (0.4)
**TOTAL**	**261**	**271**

**Table 3:** Symptoms accompanying CE as declared by patients at the time of diagnosis in the period between 2010 and 2015.

Symptom	%	Site of cyst
Abdominal pain	24	Liver (55/158), lung (9/74), intestine (1/1)
Dyspnea	13	Liver (14/158), lung (22/74)
Swollen abdomen	2.8	Liver (8/158), lung (1/74)
Appetite loss	3.3	Liver (6/158), lung (3/74)
Fever	3	Liver (4/158), lung (4/74)
Back pain	2.2	liver (4/158), Axillary region (1/1), lung (1/74)
Headache	2.2	Liver (3/158), lung (3/74)
Nervousness	1.8	Liver (4/158), lung (1/74)
Diarrhea	1.1	Liver (2/158), lung (1/74)
Weight loss	1.1	Liver (2/158), lung (1/74)
Cough	0.7	Lung (2/74)
Chills	0.7	liver (1/158), Axillary region (1/1)
Dizziness	0.7	Liver (1/158), lung (1/74)

First number in brackets indicates number of patients with symptoms over the total number of patients with cysts in the mentioned site. (%) number of patients with the symptom over the total number of cysts (n = 271)

### Molecular diagnosis and genotyping

**Fig 3:**
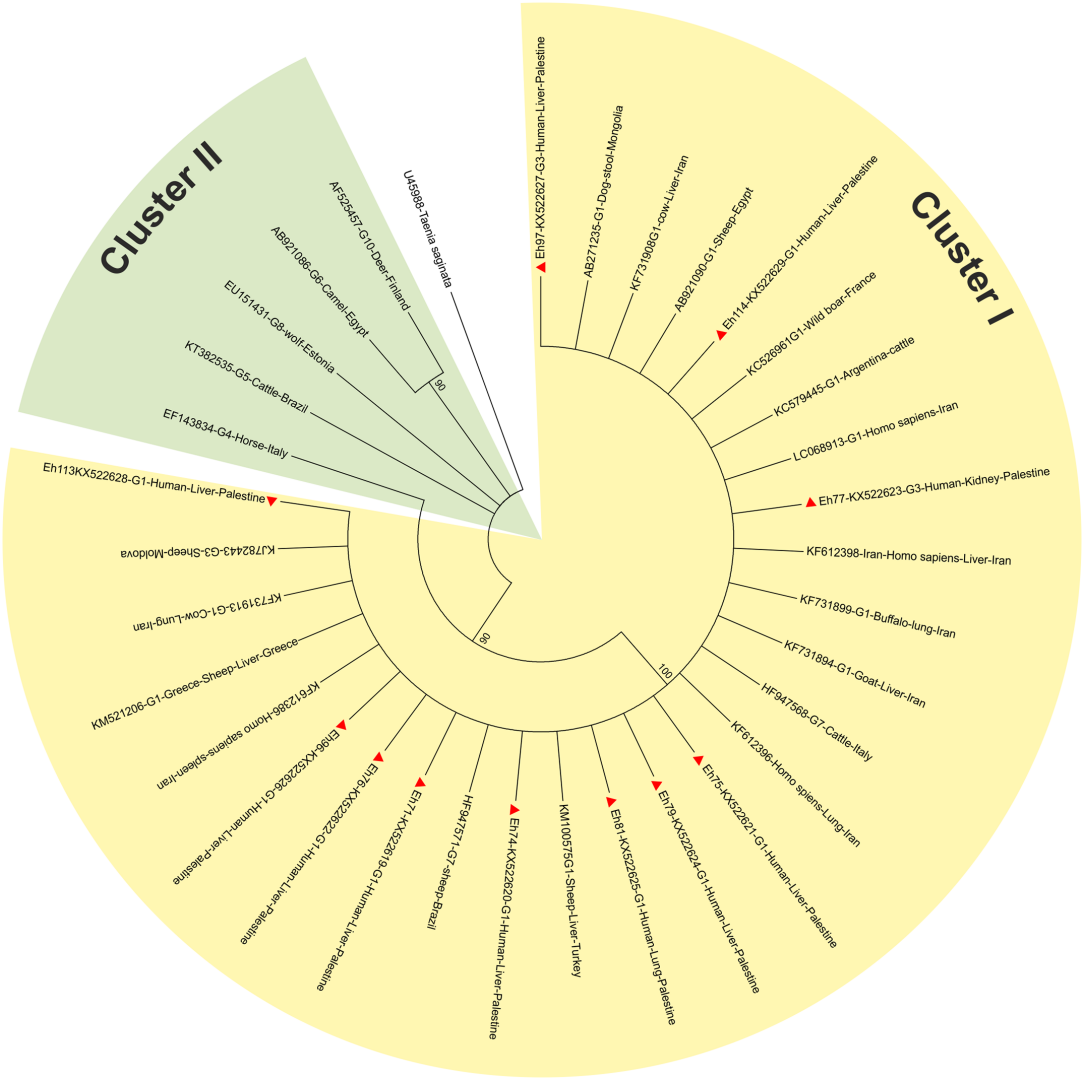
Bootstrap consensus dendrogram inferred from 1000 replicates constructed by maximum likelihood method using nucleotide sequences of the mitochondrial cytochrome *c* oxidase subunit 1 (CO 1). The analysis involved 11 nucleotide sequences collected in the study (red triangles) and 22 retrieved from Genbank in addition to *Taenia saginata* sequence, which was used as an out-group to root the tree. Sample codes shown on the dendrogram consist of the laboratory code, accession number, genotype, host, site of infection, and country of origin. Percentage of replicates is shown next to the branches.

In this study, 14 cysts were collected from patients immediately following surgery and 68 additional FFPE CE samples were collected from histopathology departments. Of these, 86% (12/14) and 82% (56/68) respectively were positive for *Echinococcus* species as identified through the amplification of the diagnostic tandem repeat product (269bp). In addition, a partial fragment of *cox 1* gene was successfully sequenced for 11 of the 14 cyst samples, which were identified using BLAST as *E*. *granulosus* s.s. (9 samples as G1 and 2 as G3 genotype). Nucleotide sequences generated here were deposited in the GenBank under the accession numbers depicted in Fig 3 showing the genetic clustering. The dendrogram showed that all 11 isolates from Palestine clustered in one group. Seven of the 353 cases were classified at hospital level as originated by *Echinococcus multilocularis* based solely on clinical picture without molecular confirmation. These 7 samples were not available in this study, thus was not possible to confirm or rule out the presence of this parasite.

## Discussion

In Palestine, despite the fact that CE is a notifiable disease, information on the magnitude of infection is usually generated by surgical wards from public government hospitals. Using this approach, an average surgical incidence of CE in the West Bank during 2010–2015 was reported to be 1.6 per 100,000 compared to the 2.1 per 100,000 generated in this study. An earlier study in which researchers scanned hospitals in the West Bank between 1990 and 1997 reported a relatively high average incidence of 3.1 per 100,000 [26]. In addition, the number of CE cases with known year of surgery included in this study was greater than that reported by the MOH in five of the six-year study period (Fig 1). A potential explanation for this discrepancy may be related to the inaccuracy in the surveillance system since the reporting of CE in official records only began in 2006, as CE was not reportable disease by law before then (Fig 1) [17]. At the same time, the Israeli health authorities reported 38 cases between 1991 and 1995 [27]. In Jordan, the surgical incidence rate between 1985 and 1993 was 2.9 per 100,000. [26, 28, 29]. Surgical incidence studies appear to underestimate the actual burden of CE. CE data collated from surgical procedures reflects the tip of the iceberg as the majority of CE cases are asymptomatic, and thus do not come to the attention of clinicians. Surgery is normally performed on symptomatic patients with complicated CE or on individuals who are inadvertently diagnosed as having the disease [30]. Furthermore, it is normal practice for patients to be referred for CE surgery to hospitals outside Palestine, such as Jordan. Those cases were not included in Palestinian annual health report. Other explanations might be due to incorrect diagnosis or underreporting by physicians or hospitals.

The distribution of CE cases by sex with slight predominance in females, but not statistically significant, is in agreement with other studies [26, 28]. Cystic echinococcosis surgical incidence was higher in the younger age groups (10–29 years). This is in congruence with other studies using surgical incidence that reported the highest number of CE cases in young patients (10–13 years) [20, 26, 28]. In rural and Bedouin areas, herds of sheep are commonly accompanied by several dogs which are in strict contact with children, increasing their exposure to this parasite. Furthermore, it’s a habit by villagers, young and adults, to eat leafy plants such as mallow (*Malva parviflora*) or those growing in the yard such as lattice, spinach and onions, thus increasing the possibility of contracting this infection.

CE has a long latency period and subsequently can be detected years after infection, which is often at an older age [31]. However, the appearance of disease among adolescents (10–20 years) Palestinian patients would suggest an infection sustained at a very early stage in life. Most of CE cases were reported in rural areas, which is in agreement with studies worldwide [29, 32, 33] and is one of the potential risk factors for acquiring CE identified in a recent systematic review for acquiring CE [34]. In contrast, it should be stressed that surgical incidence may introduce bias in the engagement of patients in this study since young age groups, especially children, could be more likely to seek medical attention compared to older age groups.

In Palestine, a study that identified high incidence of *E*. *granulosus* infection among dogs was recently published [18]. The proximity of humans to free-roaming dogs which have access to infected offal is a known potential risk factor for CE infection [34].

This study identified seventeen sites of CE infection; however 85% had a predilection for the liver and the lungs (Table 2). The liver is widely known to be the most infected organ for cystic echinococcosis in Palestine and elsewhere as a result of the portal blood flow [26, 28, 35–37]. Double-site infection was rare with mostly the liver involved, and a multiple-site infection was reported only in one case. Abdominal pain as a result of bile duct compression and dyspnea resulting from irritated lung membranes were the main symptoms reported by patients, which in turn reflects the predominance of liver and lung infections [37].

Of the 14 human CE cysts, 86% were positive for the Hae III *E*. *granulosus* repetitive gene sequence and two were negative with one having been preserved for over a month in 10% formalin, a potent DNA degrader. Similarly, the infection rate of FFPE samples was 85%. Nine (81.8%) and 2 (18.2%) of the Palestinian patients’ included in this study were molecularly confirmed as having been infected with *E*. *granulosus* G1 and G3 genotype respectively. To the best of our knowledge, this is the first molecular identification of the human CE causative agent in Palestine. *E*. *granulosus* sensu stricto (s.s.) had been previously confirmed from sheep [14] and dogs [13] and the findings of the current study point to the free circulation of this species within Palestine and demonstrate the active involvement of these hosts in the perpetuation and transmission of this parasite [18, 26]. Results obtained through the construction of the maximum likelihood tree showed *E*. *granulosus* sensu stricto (s.s.) (G1/G3) nucleotide sequences generated in this study to group within a single cluster along with *E*. *granulosus* s.s. (G1/G3) from the Genbank demonstrating genetic uniformity. This is consistent with other studies from Italy, Jordan, Iran, India, China and Peru that investigated nucleotide sequence variation of DNA extracted from CE material derived from humans, livestock and dogs confirming the low nucleotide-diverse nature of *E*. *granulosus* sensu stricto (s.s.) worldwide [18, 19, 38–41].

CE is widely spread in Palestine with the majority (94%) of cases in the West Bank and only 6% in Gaza. The low incidence in Gaza Strip may be a reflection of the total share of livestock in Palestine which is 20.2% for Gaza Strip and 79.8% for the West Bank [42]. Spatial and space-time distribution showed Al-Khalil district to be the main focus of the disease in Palestine. Al-Khalil district is the most populous district and holds the largest share of livestock animals in Palestine (21.1%) including sheep (25.2%) and goats (21%) [42]. Rural areas within Al-Khalil such as Yatta, Idhna and Dura villages appear to be the hot spots for CE; as this has been the case for the last 3 decades with an incidence of 16.8 per 100,000 in Yatta village between 1990 and 1997 (Fig 2) [26]. The seven cases of *E*. *multilocularis* identified at hospital level based only on clinical picture are the first reports in Palestine. However, in the absence of molecular confirmation, multiple cysts of *E*. *granulosus* s.s., such as CE2 according to WHO-IWGE (Informal Working Group on Echinococcosis) classification, may be erroneously identified as those of *E*. *multilocularis* [43].

In conclusion, this study has shown that *E*. *granulosus* sensu stricto (s.s.), is the most prevalent species causing human CE in the West Bank and Gaza Strip and identified Al-Khalil district as the main focus for CE infection. Risk assessment studies in the rural areas such as Yatta are a prerequisite for control measures. For this reason, we would encourage the Ministry of Health, Ministry of Agriculture, and local health authorities to implement control measures aiming at decreasing the burden of CE in humans in Palestine and interpolating a stringent surveillance system. In addition, sensitizing the Palestinian citizens by community health awareness campaigns and upgrading the level of health service by training the medical team on CE epidemiology and detection are prerequisites for effective surveillance and control of this neglected disease.
